# miR-96 promotes cell proliferation, migration and invasion by targeting PTPN9 in breast cancer

**DOI:** 10.1038/srep37421

**Published:** 2016-11-18

**Authors:** Yeting Hong, Hongwei Liang, Uzair-ur-Rehman, Yanbo Wang, Weijie Zhang, Yong Zhou, Song’an Chen, Mengchao Yu, Sufang Cui, Minghui Liu, Nan Wang, Chao Ye, Chihao Zhao, Yanqing Liu, Qian Fan, Chen-Yu Zhang, Jianfeng Sang, Ke Zen, Xi Chen

**Affiliations:** 1 State Key Laboratory of Pharmaceutical Biotechnology, NJU Advanced Institute for Life Sciences, Jiangsu Engineering Research Center for MicroRNA Biology and Biotechnology, School of Life Sciences, Nanjing University, 163 Xianlin Avenue, Nanjing, 210046, China; 2 Department of General Surgery, Affiliated Drum Tower Hospital of Nanjing University Medical School, 321 Zhongshan Road, Nanjing, 210008, China; 3 Department of Thoracic and Cardiovascular surgery, Affiliated Drum Tower Hospital of Nanjing University Medical School, 321 Zhongshan Road, Nanjing, 210008, China; 4 Key Laboratory of Cancer Prevention and Therapy, Tianjin Medical University Cancer Institute and Hospital, Huanhuxi Road, Tiyuanbei, Tianjin, 300060, China; 5 Department of Thyroid and Breast Surgery, Affiliated Drum Tower Hospital of Nanjing University Medical School, 321 Zhongshan Road, Nanjing 210008, China

## Abstract

microRNAs (miRNAs) have emerged as major regulators of the initiation and progression of human cancers, including breast cancer. The aim of this study is to determine the expression pattern of miR-96 in breast cancer and to investigate its biological role during tumorigenesis. We showed that miR-96 was significantly upregulated in breast cancer. We then investigated its function and found that miR-96 significantly promoted cell proliferation, migration and invasion *in vitro* and enhanced tumor growth *in vivo*. Furthermore, we explored the molecular mechanisms by which miR-96 contributes to breast cancer progression and identified PTPN9 (protein tyrosine phosphatase, non-receptor type 9) as a direct target gene of miR-96. Finally, we showed that PTPN9 had opposite effects to those of miR-96 on breast cancer cells, suggesting that miR-96 may promote breast tumorigenesis by silencing PTPN9. Taken together, this study highlights an important role for miR-96 in the regulation of PTPN9 in breast cancer cells and may provide insight into the molecular mechanisms of breast carcinogenesis.

Breast cancer is the most prevalent cancer in women worldwide. Although the mortality rate is decreasing, it still ranks second among the most common causes of cancer death in women^1^. Many carcinogenic factors may increase the chances of developing breast cancer, including endocrine disorders, genetic mutations and decline in immune function. However, the exact mechanisms contributing to the origin and development of breast cancer remain complex and obscure^2^.

MicroRNAs (miRNAs) are a class of 19–24 nucleotide-long non-coding RNAs that act as post-transcriptional regulators of gene expression in eukaryotes. miRNAs bind target mRNAs at complementary sites in their 3′-untranslated regions (3′-UTRs), thereby repressing translation or degrading the RNA transcripts of target genes. Recent studies have shown that miRNAs are implicated in various human cancers, including breast cancer^3^,^4^,^5^. Cancer cells show characteristic miRNA expression profiles, and miRNAs can downregulate multiple tumor suppressor genes or oncogenes during carcinogenesis, thereby functioning as oncogenes or tumor suppressors, respectively^6^. Among the miRNAs correlated with tumorigenesis, the miR-183-96-182 cluster is among the most characterized miRNAs. The miR-183-96-182 cluster is a highly conserved polycistronic miRNA cluster which was first identified in sensory organs^7^. Members of this cluster are located within a 5-kb region on human chromosome 7q32.2 and are transcribed in the same direction from telomere to centromere. Abnormal expression of the miR-183-96-182 cluster is frequently observed in a variety of cancer types, but the role of this miRNA cluster is still unclear: it may function as an oncogene or tumor suppressor gene, depending on the type, location and stage of the tumor. As a member of the miR-183-96-182 cluster, miR-96 usually functions as an oncogene during tumorigenesis. miR-96 has been shown to be overexpressed in hepatocellular carcinoma, prostate cancer, bladder cancer, lung cancer and colorectal adenocarcinoma^8^,^9^,^10^,^11^,^12^. The target genes of miR-96 include the tumor suppressor genes *FOXO1* and *FOXO3a* in breast cancer^13^,^14^, and other validated targets of miR-96 include *RECK* in esophageal cancer^15^, *EphrinA5* in Hepatocellular carcinoma^16^, *SAMD9* in non-small cell lung cancer (NSCLC)^17^. However, the molecular mechanism underlying the contribution of miR-96 to the development and progression of breast cancer remains poorly understood. The aim of this study was to evaluate the association of miR-96 with breast cancer and explore the potential novel target genes of miR-96.

In this study, we investigated miR-96 expression patterns in breast cancer and found that miR-96 levels were consistently upregulated in breast cancer tissues. Subsequently, we showed that miR-96 enhanced tumor growth in a breast cancer xenograft mouse model. Furthermore, we identified PTPN9 (protein tyrosine phosphatase, non-receptor type 9) as a direct target gene of miR-96 and showed that miR-96 inhibits PTPN9 expression and consequently promotes proliferation, migration and invasion of breast cancer cells.

## Materials and Methods

### Human tissues and cell lines

A total of 10 pairs of breast cancer and matched adjacent noncancerous tissue samples were collected between 2014 and 2015 at Nanjing Drum Tower Hospital (Nanjing, China). All protocols concerning the use of patient samples in this study were approved by the Medical Ethics Committee from Nanjing University and Nanjing Drum Tower Hospital, and all patients signed informed consent for the collection and use of their tissues for this study. The methods were carried out in accordance with the approved guidelines by Nanjing University and Nanjing Drum Tower Hospital. The clinical data of these tissues are listed in Supplementary Table 1. Two human breast cancer cell lines, MCF-7 and MDA-MB-468, and an embryonic kidney cell line, 293 T, were purchased from the Shanghai Institute of Cell Biology, Chinese Academy of Sciences (Shanghai, China). MCF-7 and 293 T cells were maintained in DMEM medium (Gibco, Carlsbad, CA, USA). MDA-MB-468 cells were maintained in 1640 medium (Gibco, Carlsbad, CA, USA). Medium was supplemented with 10% fetal bovine serum (FBS, Gibco, Carlsbad, CA, USA) and 1% penicillin/streptomycin (Gibco, Carlsbad, CA, USA). All cells were cultured in a humidified incubator at 37 °C with 5% CO_2_.

### Xenograft assays in nude mice

Four-week-old athymic BALB/c female nude (nu/nu) mice were purchased from the Model Animal Research Center of Nanjing University (Nanjing, China) and maintained under specific pathogen-free conditions at Nanjing University. The animal studies were approved by the Animal Care and Use Committee at Nanjing University. The methods were performed in accordance with the approved guidelines by Nanjing University. They were equally divided into 3 groups (6 mice/group) and injected subcutaneously with 1 × 10^7^ untreated MCF-7 cells (Mock) or MCF-7 cells infected with the control lentiviral vector (pre-miR-NC-LV) or miR-96 overexpression lentiviral vector (pre-miR-96-LV). After subcutaneous implantation of cells, animals were observed daily for tumor growth. The mice were sacrificed and photographed at 21 days post-implantation. Xenograft tumors were excised, photographed and weighed. Tumor section slides were subjected to immunohistochemical analysis using hematoxylin and eosin (H&E) staining and PCNA and Ki-67 staining according to the manufacturer’s instructions. All animal care and handling procedures were performed in accordance with the National Institutes of Health’s Guide for the Care and Use of Laboratory Animals.

### Overexpression or knockdown of miR-96

Overexpression of miR-96 was achieved by transfecting cells with miR-96 mimic (miR-96, a synthetic double-stranded RNA oligonucleotide mimicking miR-96 precursor). Knockdown of miR-96 was achieved by transfecting cells with miR-96 antisense (anti-miR-96, a chemically modified antisense oligonucleotide designed to target mature miR-96). Synthetic negative control RNAs served as controls (miR-NC and anti-miR-NC). All synthetic RNA oligonucleotides were purchased from GenePharma (Shanghai, China). MCF-7 and MDA-MB-468 cells were seeded into 6-well plates and transfected the following day when the cells were approximately 70% confluent using Lipofectamine 2000 (Invitrogen, Carlsbad, CA, USA) according to the manufacturer’s instructions. For each well, equal dose (75 pmol) of miR-NC, miR-96, anti-miR-NC or anti-miR-96 was added. Cells were harvested 24 h after transfection, and total RNA and protein were extracted for quantitative RT-PCR and western blotting analyses, respectively.

### RNA extraction and quantitative RT-PCR

Total RNA was extracted from the cell lines or human tissues using TRIzol Reagent (ambion, Carlsbad, CA, USA) according to the manufacturer’s instructions. RNA quality was determined by formaldehyde-agarose gel electrophoresis, and the concentration of RNA was determined using an Eppendorf BioPhotometer plus (Eppendorf AG, Hamburg, Germany). Assays to quantify miRNAs were performed using TaqMan miRNA probes (Applied Biosystems, Foster City, CA) according to the manufacturer’s instructions. Briefly, 2 μl (0.5 μg/μL) of total RNA was reverse-transcribed to cDNA using AMV reverse transcriptase (TaKaRa, Dalian, China) and a stem-loop RT primer (Applied Biosystems, Foster City, CA). The reaction steps were as follows: 16 °C for 30 min, 42 °C for 30 min, and 85 °C for 5 min. Real-time PCR was performed using a TaqMan PCR kit on an Applied Biosystems 7500 Sequence Detection System (Applied Biosystems, Foster City, CA). The reactions were incubated in a 96-well optical plate at 95 °C for 5 min, followed by 40 cycles of 95 °C for 15 s and 60 °C for 1 min. All reactions, including the no template controls, were run in triplicate. After the reaction finished, the C_T_ values were determined using fixed threshold settings. The relative amount of miRNA expression was normalized to U6 snRNA expression in this study by the equation 2^−ΔΔCT^, where ΔΔC_T_ = (C_T miRNA_ − C_T U6_)_target_ − (C_T miRNA_ − C_T U6_)_control_.

The expression levels of PTPN9, Cyclin D1, CDK6, CDK4, p21 and GAPDH mRNAs were determined using the SYBR Green method. Briefly, 4 μl (0.5 μg/μL) of total RNA was reverse-transcribed to cDNA using AMV reverse transcriptase (TaKaRa, Dalian, China) and Oligo d(T)18 primers (TaKaRa, Dalian, China). The reaction steps were as follows: 42 °C for 60 min and 72 °C for 10 min. Next, real-time PCR was performed with the RT products, SYBR Green dye and specific primers for each mRNAs. The sequences of primers are provided in Supplementary Table 2. All primers were synthesized by GenScript (Nanjing, China). The reactions were incubated in 96-well plates at 95 °C for 5 min followed by 40 cycles of 95 °C for 30 s, 55 °C for 30 s, and 72 °C for 30 s, followed by 1 cycle for the melt curve. All reactions were run in triplicate. When the reaction was complete, the C_T_ values were determined by setting a fixed threshold. The relative amount of each mRNA was normalized to GAPDH by the equation 2^−ΔΔCT^, where ΔΔC_T_ = (C_T PTPN9/Cyclin D1/CDK6/CDK4/p21_ − C_T GAPDH_)_target_ − (C_T gene_ − C_T GAPDH_)_control_.

### EdU proliferation assay

To assess cell proliferation, MCF-7 cells were seeded into 48-well plates. The cells were incubated under standard conditions in complete media (DMEM supplemented with 10% FBS). Transfection of the cells was performed the following day as described above. Twenty-four hours after transfection, the cells transfected with miRNA were harvested. Forty-eight hours after transfection, the cells transfected with siRNA and overexpression plasmid were harvested. Cell proliferation was detected by the incorporation of 5-ethynyl-2′-deoxyuridine (EdU) using the EdU Cell Proliferation Assay Kit (Ribobio, Guangzhou, China). Briefly, the cells were incubated with 50 μM EdU for 8 h before fixation, permeabilization and EdU staining, which were performed according to the manufacturer’s protocol. The cell nuclei were stained with Hoechst (Ribobio, Guangzhou, China). The proportion of nucleated cells incorporating EdU was determined by fluorescence microscopy.

### Wound healing assay

MCF-7 cells were seeded into 6-well plates. The cells were incubated under standard conditions in complete medium, cell transfection was performed the following day as described above, and cells were allowed to adhere for 24 h. Confluent monolayer cells were scratched by a 200 μL pipette tip and then washed three times with PBS buffer to clear cell debris and suspended cells. Fresh serum-free medium was replaced, and the cells were allowed to close the wound for 24 h. Photographs were taken at two different time points (0 and 24 h) by a light microscope (Olympus, Tokyo, Japan) at ×40 magnification. The percentage of the area with migrated cells compared to the initial wound region was defined as wound closure (setting the gap area as 0% at 0 h).

### Cell invasion assay

Transwell invasion assays were performed using Matrigel Invasion Chambers (BD Biosciences, Bedford, MA) with inserts containing an 8-μm pore-sized membrane with a thin layer of Matrigel. MCF-7 cells were transfected and harvested as mentioned above and seeded at a density of 5 × 10^6^/well on the upper chamber with serum-free DMEM. Simultaneously, 0.5 mL DMEM supplemented with 10% FBS was added to the lower compartment, and the transwell-containing plates were incubated for 8 h. At the end of the incubation, the cells that had entered the lower surface of the filter membrane were fixed with 4% paraformaldehyde for 15 min at room temperature. The cells were then washed three times with PBS buffer and stained with 0.1% crystal violet for 15 min at room temperature. The cells remaining on the upper surface of the filter membrane were gently scraped off with a cotton swab. The invaded cells were counted under microscopic observation. Each experiment was performed in triplicate and repeated twice.

### Cell cycle assay

To assess the cell cycle, MCF-7 cells were seeded into 6-well plates. The cells were incubated under standard conditions in complete media. Transfection of the cells was performed the following day as described above. 24 hours after transfection, the cells transfected with miRNA were harvested. 48 hours after transfection, the cells transfected with siRNA and overexpression plasmid were harvested. Cells were washed twice with PBS and fixed in 75% ethanol overnight. Then, cells were washed twice with PBS buffer and 50 μg/ml RNase A for 30 min at 37 °C. Staining for DNA content was performed using 50 mg/ml propidium iodide (BD Biosciences, San Jose, CA). Analysis was performed on a fluorescence-activated cell-sorting (FACS) flow cytometer (BD Biosciences, San Jose, CA) with Cell Quest Pro software. Cell cycle modeling was performed with Flowjo software.

### miRNA target prediction

The miRNAs that may target PTPN9 were determined using algorithms from TargetScan (http://genes.mit.edu/targetscan/), PicTar (http://pictar.bio.nyu.edu/), and miRanda (http://cbio.mskcc.org/cgi-bin/mirnaviewer/mirnaviewer.pl).

### Luciferase assay

To construct a luciferase reporter carrying the PTPN9 3′UTR with a predicted potential binding site of miR-96, we amplified a 1608 bp PTPN9 3′UTR region from the genomic DNA using the following PCR primers: PTPN9-3′UTR (sense): 5′-GGACTAGTCTCTCCTACGAACCTCCTAC-3′; and PTPN9-3′UTR (antisense): 5′-CGACGCGTCTGTATCACTGTAAGATATTG-3′. The amplified fragment was cloned into the pMIR-Report plasmid (Ambion, Austin, TX, USA) at the *Spe* I & *Mlu* I site. We also constructed a pMIR-Report plasmid that carried the mutant PTPN9 3′UTR region. For the luciferase reporter assays, 293 T cells were cultured in 24-well plates, and each well was transfected with 0.3 μg firefly luciferase reporter plasmid, 0.15 μg β-galactosidase expression vector (Ambion, Austin, TX, USA), and equal amounts of miR-NC or miR-96 using Lipofectamine 2000 (Invitrogen). The β-galactosidase vector was used as transfection control. Cells were assayed 24 h after transfection using luciferase assay kits (Promega, Madison, WI, USA).

### Plasmid construction and siRNA interference assay

Two siRNAs (siRNA-I and siRNA-II) targeting human PTPN9 cDNA were designed and synthesized by Ribobio (Guangzhou, China). A scrambled siRNA served as a negative control (siRNA-NC). A mammalian expression plasmid (pReceiver-M02-PTPN9) designed to encode the full-length open reading frame (ORF) of human PTPN9 without the 3′-UTR was purchased from GeneCopoeia (Germantown, MD, USA). An empty plasmid (pReceiver-M02) served as a negative control (plasmid-NC). The overexpression plasmid or siRNA of PTPN9 were transfected into MCF-7 and MDA-MB-468 cells using Lipofectamine 2000 (Invitrogen) according to the manufacturer’s instructions. Total RNA or protein was isolated 48 h after transfection. The mRNA and protein expression levels of PTPN9 were assessed by quantitative RT-PCR and western blotting, respectively.

### Protein extraction and western blotting

Cells were rinsed with PBS (pH 7.4) and then lysed in RIPA lysis buffer (Beyotime, Shanghai, China) with freshly added PMSF (Beyotime, Shanghai, China) for 30 min on ice. Tissue samples were frozen solid with liquid nitrogen, ground into a powder and lysed in RIPA lysis buffer supplemented with PMSF on ice for 30 min. Sonication was used to facilitate cell lysis. After centrifugation at 16,000 g, 4 °C for 10 min, the supernatants were collected and the protein concentration was quantified using a BCA protein assay kit (Thermo Scientific, Rockford, IL, USA). The protein levels were quantified by western blotting analysis of cell extracts or tissue extracts using antibodies below: anti-PTPN9 (MAB2668) was purchased from R&D Systems Inc. (Minneapolis, USA); anti-CDK4 (12790) and anti-p21 (2947) were purchased from Cell Signaling (Danvers, MA, USA); anti-Cyclin D1 (ab 134175) and anti-CDK6 (ab 124821) were purchased from Abcam (Cambridge, MA, USA). These proteins levels were normalized by probing the same blots with an anti-GAPDH antibody (sc-47724) purchased from Santa Cruz (Dallas, TX, USA).

### Statistical analysis

All images of western blotting and the EdU proliferation assay are representative of at least three independent experiments. Quantitative RT-PCR and the luciferase reporter assay were performed in triplicate, and each experiment was repeated several times. The data shown are the mean ± SE of at least three independent experiments. Statistical analysis was performed using Student’s t-test, and the data were considered significant if the *p* value was <0.05 (indicated by *), <0.01 (indicated by **) or <0.001 (indicated by ***).

## Results

### miR-96 is upregulated in breast cancer tissues

**Figure 1:**
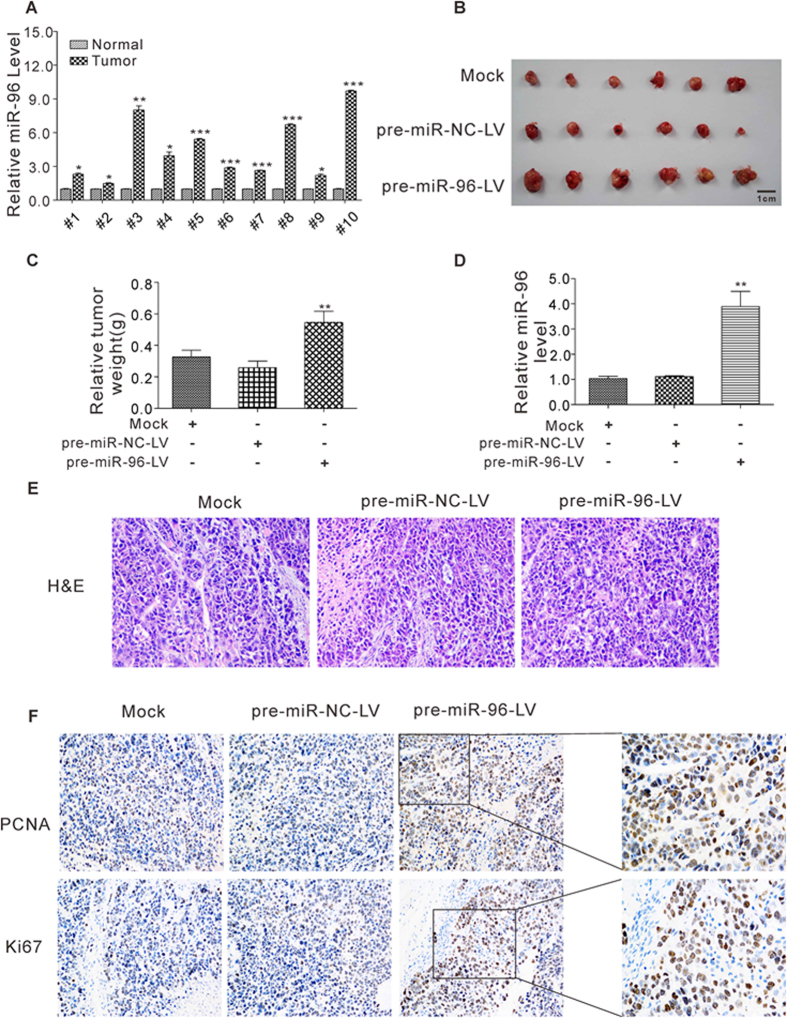
Expression levels of miR-96 in breast cancer tissues and xenograft tumors in mice. (**A**) Quantitative RT-PCR analysis of the relative expression levels of miR-96 in 10 pairs of breast cancer tissues and matched adjacent noncancerous tissues. (**B** and **C**) MCF-7 cells were infected with a control lentivirus (pre-miR-NC-LV) or a lentivirus to overexpress miR-96 (pre-miR-96-LV) and then implanted subcutaneously into 4-week-old nude mice. Tumor growth was evaluated at day 21 after cell implantation. Mice implanted with wide-type MCF-7 cells (Mock) serve as the negative control. (**B**) Representative images of the nude mice excised tumors; (**C**) Relative tumor weight. (**D**) Quantitative RT-PCR analysis of miR-96 levels in the tumors from implanted mice. (**E**) Representative H&E-stained sections of the tumors from implanted mice. (**F**) Proliferative activity assessed by anti-PCNA and anti-Ki-67 monoclonal antibody in the tumors from implanted mice. *P < 0.05; **P < 0.01; ***P < 0.001.

First, we determined the expression patterns of miR-96 in human breast cancer tissues. After measuring the expression levels miR-96 in 10 pairs of breast cancer tissues and adjacent noncancerous tissues, miR-96 levels were found to be consistently increased in breast cancer tissues compared to noncancerous tissues (Fig. 1A).

### miR-96 functions as an oncogenic miRNA in breast cancer progression

We next evaluated the biological effects of miR-96 on breast tumorigenesis in a breast cancer xenograft mouse model. MCF-7 cells were infected with a lentiviral expression vector to overexpress miR-96 and then implanted subcutaneously into 4-week-old nude mice. Tumor growth was evaluated at day 21 after cell implantation. A significant increase in the size and weight of the tumors was observed in the miR-96-overexpressing group compared to control group (Fig. 1B and C and Supplementary Figure 1). Subsequently, total RNA was extracted from each tumor and used to evaluate the expression levels of miR-96. After 21 days of xenograft growth *in vivo*, tumors from the miR-96-overexpressing group showed a significant increase in miR-96 expression compared to tumors from the control group (Fig. 1D). Furthermore, tumor tissues were embedded in paraffin and then stained with H&E for histology examination. The results revealed more cell mitosis in the miR-96-overexpressing group compared to the control group (Fig. 1E). Finally, the proliferative activity of the tumor cells was assessed via immunohistochemical staining of Ki-67 and PCNA. The tumor cell proliferation rate, as measured by the staining intensity of Ki-67 and PCNA, was increased in tumors from the miR-96-overexpressing group (Fig. 1F).

**Figure 2:**
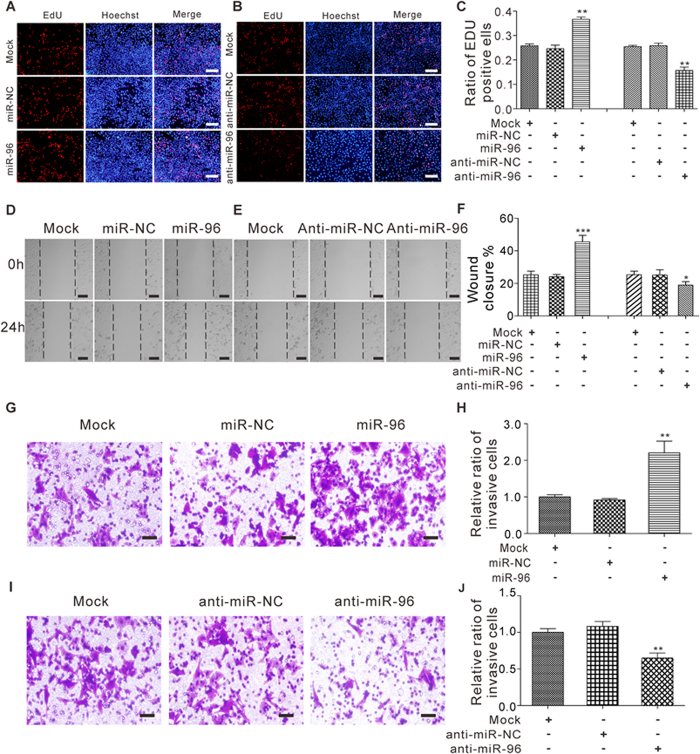
The effect of miR-96 on the proliferation, migration and invasion of breast cancer cells. (**A–C**) The EdU proliferation assay was performed 24 h after the transfection of MCF-7 cells with equal doses of miR-NC, miR-96, anti-miR-NC or anti-miR-96. The untransfected cells (Mock) serve as the negative control. The cells with red fluorescence are in the S phase of mitosis, and the cells with blue fluorescence represent all of the cells. (**A** and **B**) representative images, Scale bar = 300 μm; (**C**) quantitative analysis of EdU-positive MCF-7 cells. (**D–F**) Cell migration ability was analyzed using wound healing assays after the transfection of MCF-7 cells with equal doses of miR-NC, miR-96, anti-miR-NC or anti-miR-96. (**D** and **E**) Representative images, Scale bar = 100 μm; (**F**) quantitative analysis s of wound closure. (**G–J**) Cell invasion ability was analyzed using transwell assays after the transfection of MCF-7 cells with equal doses of miR-NC, miR-96, anti-miR-NC or anti-miR-96. (**G** and **I**) Representative images, Scale bar = 250 μm; (**H** and **J**) quantitative analysis. *P < 0.05; **P < 0.01; ***P < 0.001.

Subsequently, we ectopically expressed miR-96 in the MCF-7 human breast cancer cell line by transfecting cells with miR-96 mimic and then examined the effects of miR-96 on cell proliferation using the EdU assay. The efficient overexpression of miR-96 in MCF-7 cells is shown in Supplementary Figure 2A. In support of the notion that miR-96 functions as an oncogenic miRNA^2^,^14^,^18^,^19^,^20^, the cell proliferation rate, as measured by the percentage of EdU-positive cells, was significantly increased in MCF-7 cells transfected with miR-96 mimic (Fig. 2A and C). We also knocked down miR-96 in MCF-7 cells with miR-96 antisense. The efficient knockdown of miR-96 in MCF-7 cells is shown in Supplementary Figure 2B. As expected, the percentage of EdU-positive cells significantly decreased in MCF-7 cells transfected with miR-96 antisense (Fig. 2B and C). Moreover, wound healing assay was applied to detect migration of transfected MCF-7 cells. Overexpression of miR-96 increased cell motility while inhibition of miR-96 suppressed cell motility (Fig. 2D–F). Meanwhile, we assessed the role of miR-96 in regulating cell invasion using the transwell assay. The percentage of invaded cells was significantly higher in MCF-7 cells transfected with miR-96 mimic (Fig. 2G and H). In contrast, knockdown of miR-96 had an opposite effect on the cell invasion ability in MCF-7 cells (Fig. 2I and J). In summary, these results suggested that miR-96 may function as an oncomiR and promote cell proliferation, migration and invasion during breast cancer progression.

**Figure 3:**
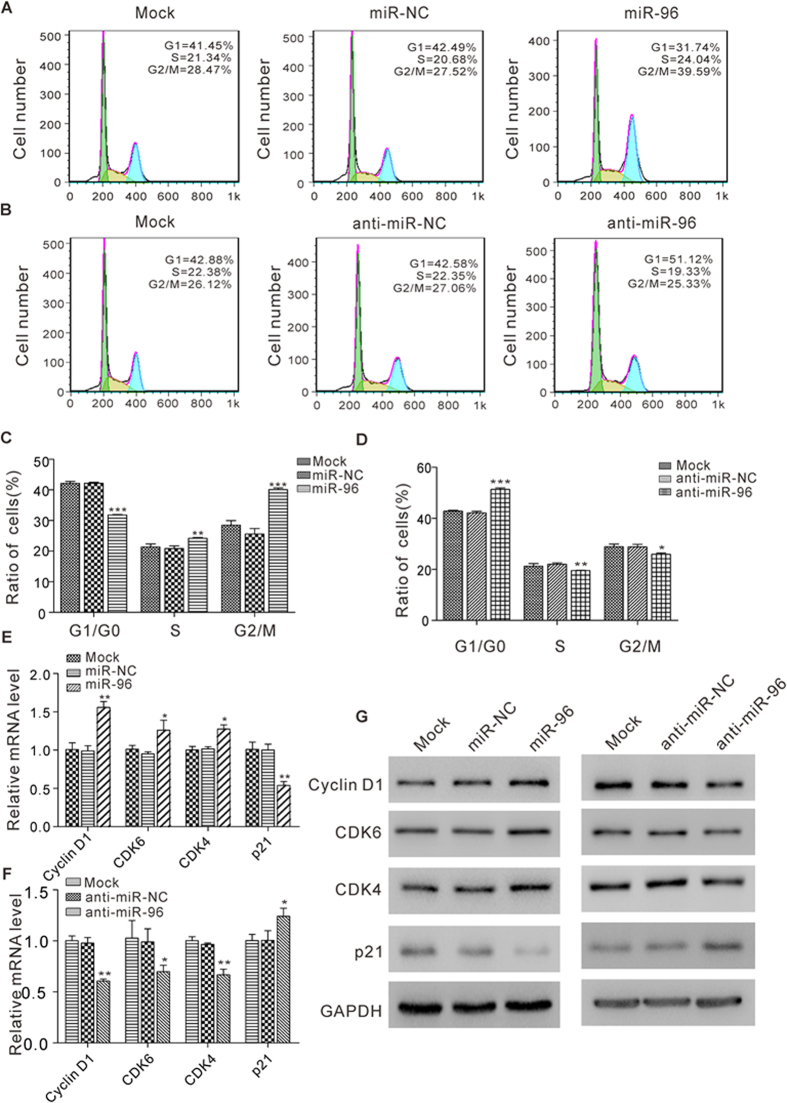
The effect of miR-96 on cell cycle progression in breast cancer cells. (**A–D**) Cell cycle profiles were analyzed using flow cytometry after the transfection of MCF-7 cells with equal doses of miR-NC, miR-96, anti-miR-NC or anti-miR-96. The untransfected cells (Mock) serve as the negative control. The panel shows histograms of cell numbers (y axis) against DNA content (x axis) determined by measuring fluorescence intensity. Numbers denote the percentages of cells in the G1/G0, S and G2/M phases. (**A** and **B**) Representative images; (**C** and **D**) quantitative analysis. (**E** and **F**) Quantitative RT-PCR analysis of the relative expression levels of Cyclin D1, CDK6, CDK4 and p21 mRNA in MCF-7 cells transfected with equal doses of miR-NC, miR-96, anti-miR-NC or anti-miR-96. The untransfected cells (Mock) serve as the negative control. (**G**) Western blot analysis of Cyclin D1, CDK6, CDK4 and p21 protein levels in MCF-7 cells transfected with equal doses of miR-NC, miR-96, anti-miR-NC or anti-miR-96. The untransfected cells (Mock) serve as the negative control. *P < 0.05; **P < 0.01; ***P < 0.001.

To further investigate the mechanism through which miR-96 promotes cell proliferation, we determined the effects of miR-96 on cell cycle regulation. For this purpose, MCF-7 cells were transfected with miR-96 mimic or antisense, and then the cell cycle status was analyzed using flow cytometry. MCF-7 cells transfected with miR-96 mimic showed a reduction of cells in the G0/G1 stage, whereas the numbers of cells in the S and G2/M phases increased (Fig. 3A and C). In contrast, MCF-7 cells transfected with anti-miR-96 showed an accumulation of cells in the G0/G1 stage, whereas the numbers of cells in the S and G2/M phases decreased (Fig. 3B and D). Cell division relies on the activation of cyclins, which bind to cyclin-dependent kinases (CDKs) to induce cell-cycle progression towards S phase and, later, to initiate mitosis^21^,^22^,^23^. The activity of CDKs can be blocked by natural CDK inhibitors, such as p21^24^,^25^. We next checked the effects of miR-96 on the cell cycle regulators (Cyclin D1, CDK6, CDK4 and p21) using quantitative RT-PCR and western blot analysis. miR-96 overexpression caused increased mRNA and protein levels of Cyclin D1, CDK6 and CDK4 in MCF-7 cells, while suppression of miR-96 resulted in decreased mRNA and protein levels of Cyclin D1, CDK6 and CDK4 (Fig. 3E–G). In contrast, overexpression of miR-96 reduced p21 mRNA and protein levels in MCF-7 cells as compared to controls, while inhibition of miR-96 increased p21 at both mRNA and protein levels in MCF-7 cells (Fig. 3E–G). Thus, it can be concluded that miR-96 may have a role in enhanced cell cycle progression in breast cancer cells, while downregulation of miR-96 is associated with cell cycle arrest and suppression of the cell cycle regulators of cyclins and CDKs.

### Prediction of PTPN9 as a target gene of miR-96

**Figure 4:**
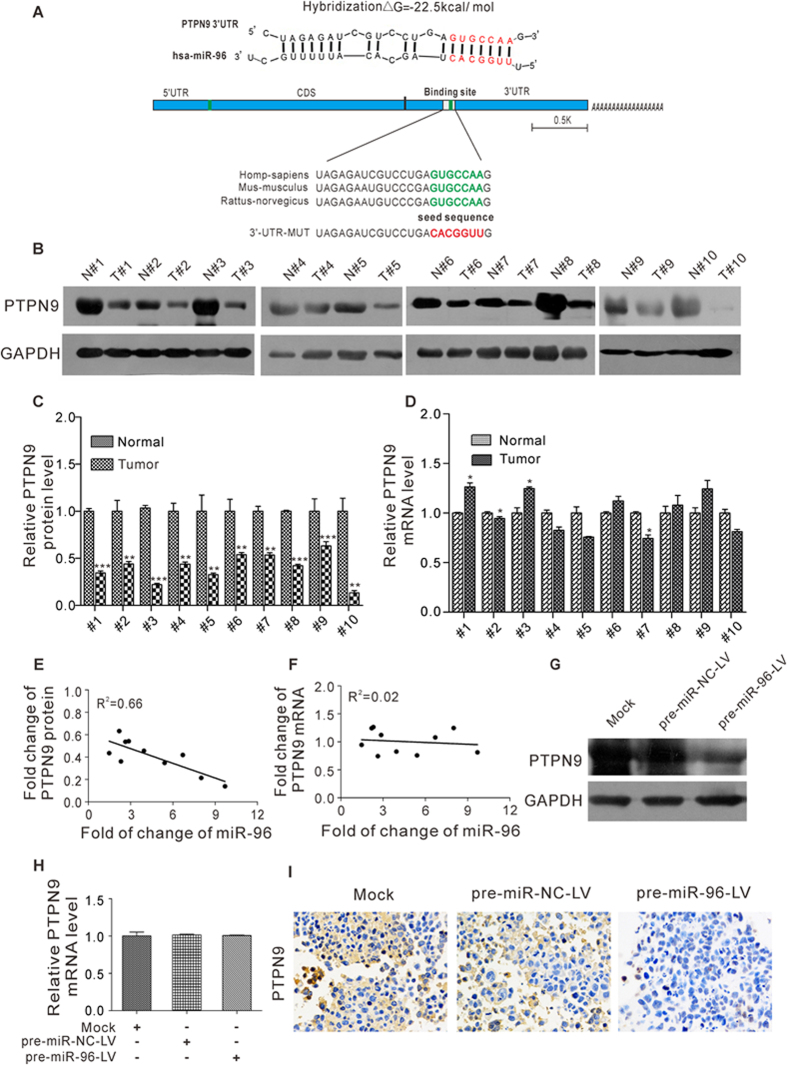
PTPN9 was predicted as a target of miR-96 and was downregulated in breast cancer tissues. (**A**) Schematic description of the hypothesized duplex formed by interaction between the PTPN9 3′-UTR binding site and miR-96. The predicted free energy value of the hybrid is indicated. The seed recognition site is denoted, and all nucleotides in this region are highly conserved across species, including human, mouse and rat. (**B** and **C**) Western blotting analysis of the expression levels of PTPN9 protein in 10 pairs of breast cancer tissues and matched adjacent noncancerous tissues. (**B**) Representative image; (**C**) quantitative analysis. (**D**) Quantitative RT-PCR analysis of the relative expression levels of PTPN9 mRNA in 10 pairs of breast cancer tissues and matched adjacent noncancerous tissues. (**E**) Pearson’s correlation scatter plot of the fold-change of miR-96 and PTPN9 protein in human breast cancer tissues. (**F**) Pearson’s correlation scatter plot of the fold change of miR-96 and PTPN9 mRNA in human breast cancer tissues. (**G–I**) MCF-7 cells were infected with a control lentivirus (pre-miR-NC-LV) or a lentivirus to overexpress miR-96 (pre-miR-96-LV) and then implanted subcutaneously into 4-week-old nude mice. Tumor growth was evaluated at day 21 after cell implantation. Mice implanted with wide-type MCF-7 cells (Mock) serve as the negative control. (**G**) Western blotting analysis of PTPN9 protein levels in the tumors from implanted mice. (**H**) Quantitative RT-PCR analysis of PTPN9 mRNA levels in the tumors from implanted mice. (**I**) Immunohistochemical analysis of PTPN9 protein levels in the tumors from implanted mice. *P < 0.05; **P < 0.01; ***P < 0.001.

To explore the molecular mechanism by which miR-96 contributes to breast cancer progression, three computational algorithms including TargetScan^26^, miRanda^27^ and PicTar^28^ were used in combination to search for potential targets of miR-96. Among the candidates, PTPN9, a tumor suppressor gene that is frequently downregulated in breast cancer^29^,^30^, was predicted to be a miR-96 target by all three of the algorithms and was selected for further experimental verification. The predicted interaction between miR-96 and the target site in the PTPN9 3′-UTR is illustrated in Fig. 4A. The 3′-UTR of PTPN9 contains one conserved binding site for miR-96. There was perfect base-pairing between the seed region (the seed sequence that encompasses the first 2–8 bases from the mature miRNA 5′end) and the cognate target. The minimum free energy value of the hybrid was −22.5 kcal/mol, which is well within the range of genuine miRNA-target pairs (Fig. 4A).

Because miRNAs are generally thought to have expression patterns that are opposite to that of their targets, we next investigated whether miR-96 expression is inversely correlated with PTPN9 expression in breast cancer. We measured the expression levels of PTPN9 in the same 10 pairs of breast cancer tissues and corresponding noncancerous tissues and found that PTPN9 protein levels were consistently lower in the cancer tissues (Fig. 4B and C). In contrast, PTPN9 mRNA levels did not differ significantly between the cancerous and noncancerous tissues (Fig. 4D), which is in accordance with a post-transcriptional mechanism that is involved in the regulation of PTPN9. The inverse correlation between miR-96 and PTPN9 protein levels (Fig. 4E) and the disparity between the miR-96 and PTPN9 mRNA levels (Fig. 4F) were further illustrated using Pearson’s correlation scatter plots. Meanwhile, we found similar results in tumors of xenograft mice. Tumors from the group with miR-96 overexpression displayed reduced PTPN9 protein levels, but not mRNA levels, compared to tumors from the control group (Fig. 4G and H). Immunohistochemical staining also revealed the presence of lower levels of PTPN9 in the group implanted with miR-96-overexpressing cells (Fig. 4I). Thus, PTPN9 was denoted as a target of miR-96 based on both computational predictions and the inverse correlation between miR-96 and PTPN9 protein levels in human breast cancer tissues.

### Validation of PTPN9 as a direct target of miR-96

**Figure 5:**
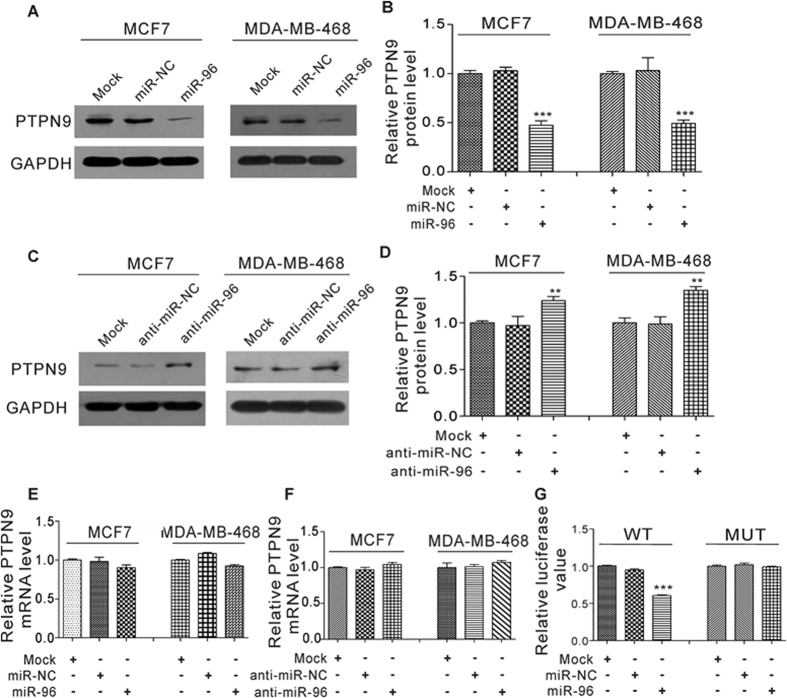
PTPN9 is a direct target of miR-96. (**A–D**) Western blot analysis of PTPN9 protein levels in MCF-7 and MDA-MB-468 cells transfected with equal doses of miR-NC, miR-96, anti-miR-NC or anti-miR-96. The untransfected cells (Mock) serve as the negative control. (**A** and **C**) Representative image; (**B** and **D**) Quantitative analysis. (**E** and **F**) Quantitative RT-PCR analysis of the relative expression levels of PTPN9 mRNA in MCF-7 and MDA-MB-468 cells transfected with equal doses of miR-NC, miR-96, anti-miR-NC or anti-miR-96. The untransfected cells (Mock) serve as the negative control. (**G**) Direct recognition of the PTPN9 3′-UTR by miR-96. Firefly luciferase reporters containing wild-type (WT) or mutant (MUT) miR-96 binding sites in the PTPN9 3′-UTR were co-transfected into 293 T cells with equal doses of miR-NC or miR-96. The cells transfected only with luciferase reporters (Mock) serve as the negative control. Twenty-four hours after transfection, luciferase assays were performed. Firefly luciferase values were normalized to β-galactosidase activity and the results were calculated as the ratio of firefly luciferase activity in the transfected cells normalized to the mock cells. **P < 0.01; ***P < 0.001.

The correlation between miR-96 and PTPN9 was further examined by evaluating PTPN9 expression levels in two human breast cancer cell lines, MCF-7 and MDA-MB-468, after overexpression or knockdown of miR-96. The efficient overexpression or knockdown of miR-96 in MDA-MB-468 cells is shown in Supplementary Figure 2. The expression of PTPN9 protein was significantly reduced by the introduction of miR-96 in MCF-7 and MDA-MB-468 cells (Fig. 5A and B), while miR-96 antisense significantly increased the PTPN9 protein levels in MCF-7 and MDA-MB-468 cells (Fig. 5C and D). Moreover, overexpression or knockdown of miR-96 did not affect PTPN9 mRNA levels (Fig. 5E and F).

To confirm that miR-96 directly targets the presumed binding sites in the PTPN9 3′-UTR and negatively regulates PTPN9 expression, a luciferase reporter assay was performed. The PTPN9 3′-UTR containing the presumed miR-96 binding site was fused downstream of the firefly luciferase gene in a reporter plasmid. The recombination plasmid was transfected into 293 T cells along with miR-96 mimic. As expected, overexpression of miR-96 resulted in ~40% reduction of luciferase reporter activity (Fig. 5G). Meanwhile, we introduced a point mutation into the corresponding complementary site in the PTPN9 3′-UTR to eliminate the predicted miR-96 binding site. The mutated luciferase reporter was unaffected by overexpression of miR-96 (Fig. 5G). These results suggests that the binding site strongly contributes to the miRNA:mRNA interaction and mediates the post-transcriptional repression of PTPN9 expression. To validate that the luciferase activity was specifically affected by miR-96, but was not other artificial effects caused by miR-96 overexpression, anti-miR-96 was added along with miR-96 in a dose dependent manner to neutralize miR-96. As expected, miR-96-induced reduction of luciferase reporter activity was gradually restored by increasing anti-miR-96 input (Supplementary Figure 3). In conclusion, the results demonstrate that miR-96 directly recognizes and binds to the 3′-UTR of the PTPN9 mRNA transcript and inhibits PTPN9 translation.

### PTPN9 has opposite effects of miR-96 on breast cancer cells

**Figure 6:**
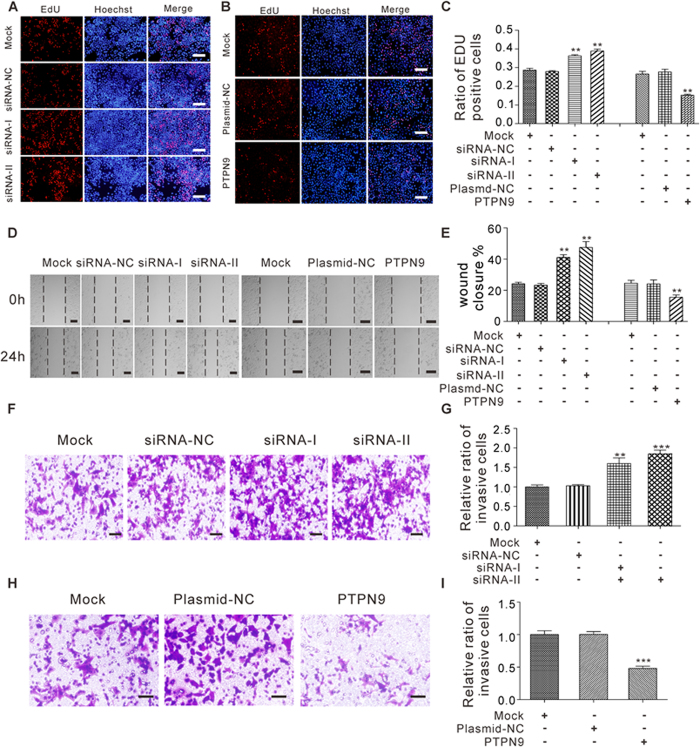
The effect of PTPN9 on the proliferation, migration and invasion of breast cancer cells. (**A–C**) The EdU proliferation assay was performed 48 h after the transfection of MCF-7 cells with equal doses of siRNA-NC or PTPN9 siRNA, or with equal doses of plasmid-NC or PTPN9 overexpression plasmid. The untransfected cells (Mock) serve as the negative control. The cells with red fluorescence are in the S phase of mitosis, and the cells with blue fluorescence represent all of the cells. (**A** and **B**) Representative images, Scale bar = 300 μm; (**C**) quantitative analysis of EdU-positive MCF-7 cells. (**D** and **E**) Cell migration ability was analyzed using wound healing assays after the transfection of MCF-7 cells with equal doses of siRNA-NC or PTPN9 siRNA, or with equal doses of plasmid-NC or PTPN9 overexpression plasmid. (**D**) Representative images, Scale bar = 100 μm; (**E**) quantitative analysis s of wound closure. (**F–I**) Cell invasion ability was analyzed using transwell assays after the transfection of MCF-7 cells with equal doses of siRNA-NC or PTPN9 siRNA, or with equal doses of plasmid-NC or PTPN9 overexpression plasmid. The untransfected cells (Mock) serve as the negative control. (**F** and **H**) representative images, Scale bar = 250 μm; (**G** and **I**) quantitative analysis. **P < 0.01; ***P < 0.001.

To investigate whether miR-96 may promote breast tumorigenesis by silencing PTPN9, we assessed the role of PTPN9 on cell proliferation, migration, invasion and cell cycle status after overexpression or knockdown of PTPN9 in breast cancer cells. To knock down PTPN9, two siRNA sequences targeting different sites of the human PTPN9 open reading frame (ORF) were designed. For overexpression of PTPN9, a plasmid designed to specially express the full-length ORF of PTPN9 without the miR-96-responsive 3′-UTR was constructed. The efficient overexpression or knockdown of PTPN9 in MCF-7 cells is shown in Supplementary Figure 4. Transfecting PTPN9 siRNAs markedly increased the percentage of proliferative EdU-positive cells, whereas transfecting the PTPN9-overexpression plasmid decreased cell proliferation (Fig. 6A–C). Likewise, transfection of PTPN9 siRNAs promoted cell migration in wound healing assay, while transfection of PTPN9-overexpression plasmid repressed cell migration (Fig. 6D and E). Additionally, transfection of PTPN9 siRNAs markedly increased the number of MCF-7 cells that passed through the transwell chamber, whereas transfection of the PTPN9-overexpression plasmid significantly reduced invasion ability (Fig. 6F–I). Because miR-96 and its target PTPN9 had opposite expression patterns and biological functions in breast cancer cells, it is quite possible that miR-96 may promote breast tumorigenesis by silencing PTPN9.

**Figure 7:**
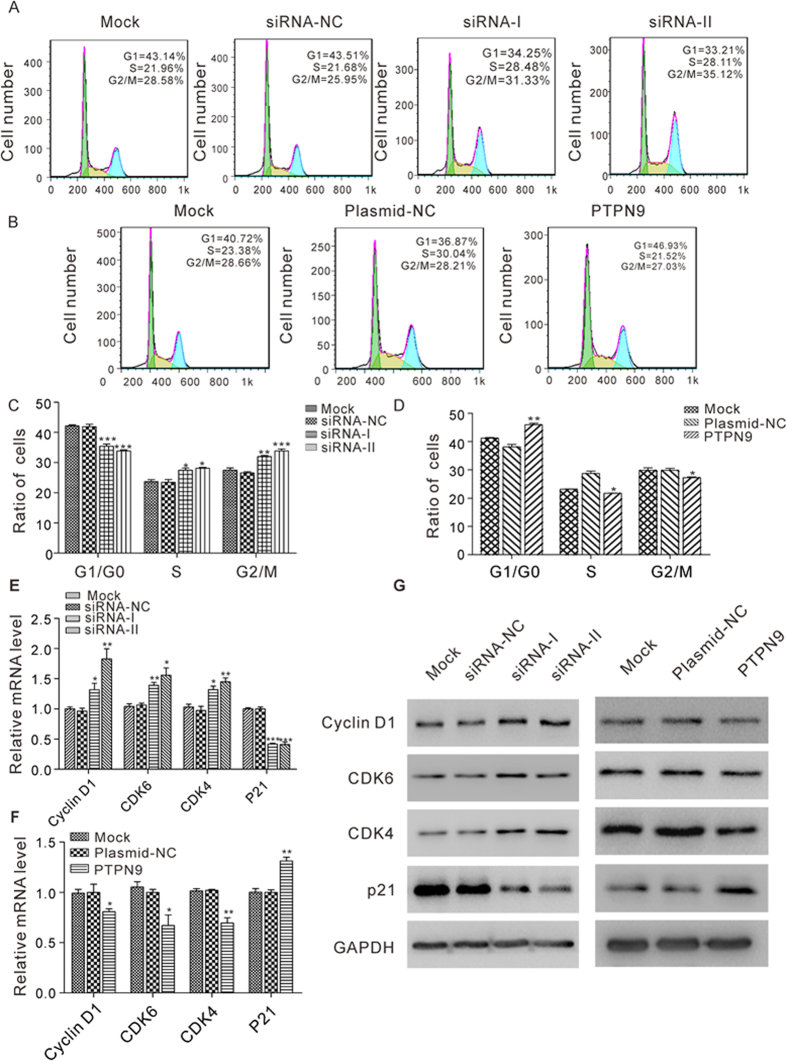
The effect of PTPN9 on cell cycle progression in breast cancer cells. (**A–D**) Cell cycle profiles were analyzed using flow cytometry after the transfection of MCF-7 cells with equal doses of siRNA-NC or PTPN9 siRNA, or with equal doses of plasmid-NC or PTPN9 overexpression plasmid. The untransfected cells (Mock) serve as the negative control. The panel shows histograms of cell numbers (y axis) against DNA content (x axis) determined by measuring fluorescence intensity. Numbers denote the percentages of cells in the G1/G0, S and G2/M phases. (**A** and **B**) Representative images; (**C** and **D**) quantitative analysis. (**E** and **F**) Quantitative RT-PCR analysis of the relative expression levels of Cyclin D1, CDK6, CDK4 and p21 mRNA in MCF-7 cells transfected with equal doses of siRNA-NC or PTPN9 siRNA, or with equal doses of plasmid-NC or PTPN9 overexpression plasmid. The untransfected cells (Mock) serve as the negative control. (**G**) Western blot analysis of Cyclin D1, CDK6, CDK4 and p21 protein levels in MCF-7 cells transfected with equal doses of siRNA-NC or PTPN9 siRNA, or with equal doses of plasmid-NC or PTPN9 overexpression plasmid. The untransfected cells (Mock) serve as the negative control. *P < 0.05; **P < 0.01; ***P < 0.001.

Next, we analyzed cell cycle profiles using flow cytometry after the transfection of MCF-7 cells with PTPN9 siRNAs or PTPN9-overexpression plasmid. The numbers of cells accumulated in the G0/G1 phase were decreased while those in S and G2/M phases were increased when transfecting with PTPN9 siRNAs (Fig. 7A and C). In contrast, overexpression of PTPN9 increased cells in the G0/G1 stage but decreased cells in the S and G2/M phases (Fig. 7B and D). Furthermore, we investigated the effects of PTPN9 on cell cycle regulators. Cyclin D1, CDK6 and CDK4 were significantly upregulated at both mRNA and protein levels in MCF-7 cells tranfected with PTPN9 siRNAs, while p21 mRNA and protein levels were downregulated after silencing PTPN9 in MCF-7 cells (Fig. 7E and G). In contrast, overexpression of PTPN9 caused decreased mRNA and protein levels of Cyclin D1, CDK6 and CDK4 and increased mRNA and protein levels of p21 in MCF-7 cells (Fig. 7F and G). Thus, miR-96 and PTPN9 may have opposite effects on cell cycle regulation in breast cancer cells.

**Figure 8:**
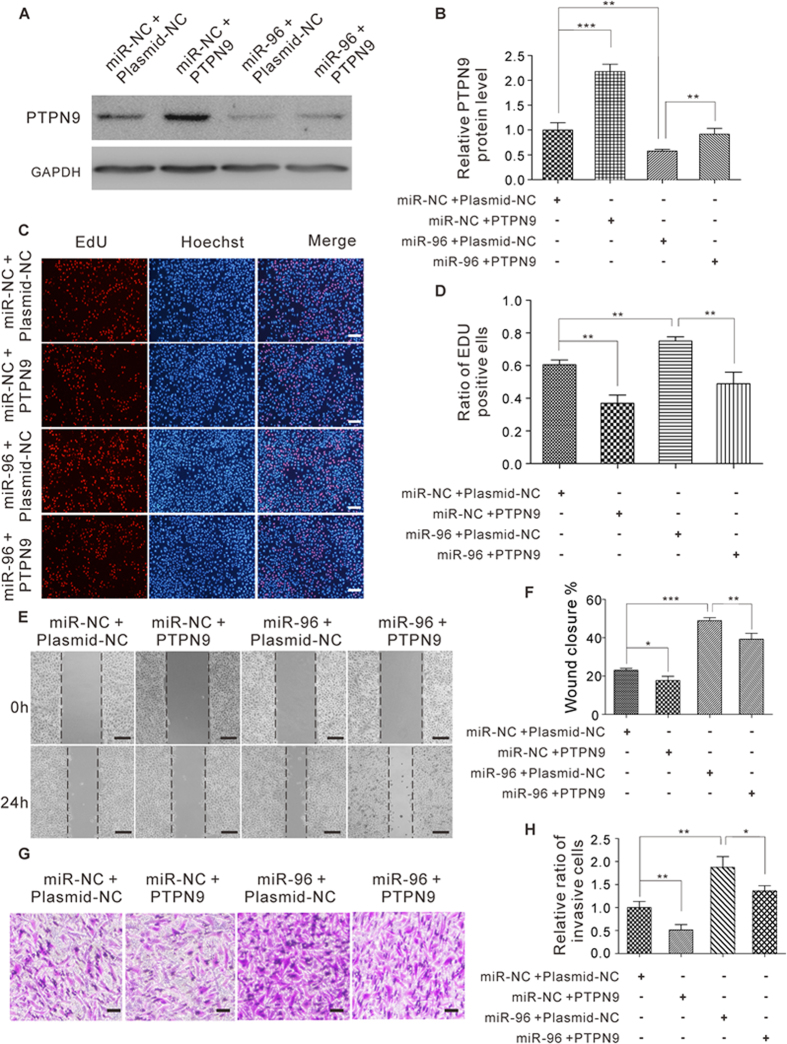
Co-effect of miR-96 and PTPN9 on the proliferation, migration and invasion of breast cancer cells. (**A** and **B**) Western blot analysis of PTPN9 protein levels in MCF-7 cells transfected with equal doses of miR-NC plus plasmid-NC, miR-NC plus PTPN9 overexpression plasmid, miR-96 plus plasmid-NC, or miR-96 plus PTPN9 overexpression plasmid. (**A**) Representative image; (**B**) quantitative analysis. (**C** and **D**) The EdU proliferation assay was performed 24 h after the transfection of MCF-7 cells with equal doses of miR-NC plus plasmid-NC, miR-NC plus PTPN9 overexpression plasmid, miR-96 plus plasmid-NC, or miR-96 plus PTPN9 overexpression plasmid. The cells with red fluorescence are in the S phase of mitosis, and the cells with blue fluorescence represent all of the cells. (**C**) Representative images, Scale bar = 500 μm; (**D**) quantitative analysis of EdU-positive MCF-7 cells. (**E** and **F**) Cell migration ability was analyzed using wound healing assays after the transfection of MCF-7 cells with equal doses of miR-NC plus plasmid-NC, miR-NC plus PTPN9 overexpression plasmid, miR-96 plus plasmid-NC, or miR-96 plus PTPN9 overexpression plasmid. (**E**) Representative images, Scale bar = 200 μm; (**F**) quantitative analysis s of wound closure. (**G** and **H**) Cell invasion ability was analyzed using transwell assays after the transfection of MCF-7 cells with equal doses of miR-NC plus plasmid-NC, miR-NC plus PTPN9 overexpression plasmid, miR-96 plus plasmid-NC, or miR-96 plus PTPN9 overexpression plasmid. (**G**) Representative images, Scale bar = 250 μm; (**H**) quantitative analysis. *P < 0.05; **P < 0.01; ***P < 0.001.

To investigate whether the regulation of cell proliferation, migration and invasion by miR-96 is executed through a PTPN9-dependent manner, we co-transfected MCF-7 cells with miR-96 mimic and the PTPN9-overexpression plasmid. Compared with cells transfected with miR-96 mimic and control plasmid, the cells transfected with both miR-96 mimic and the PTPN9-overexpression plasmid exhibited a higher PTPN9 protein level (Fig. 8A and B), suggesting that miR-96-resistant PTPN9 is sufficient to rescue the suppression of PTPN9 by miR-96. Consequently, the cells transfected with both miR-96 mimic and PTPN9-overexpression plasmid exhibited a significantly lower proliferation rate (Fig. 8C and D), suggesting that miR-96-resistant PTPN9 can attenuate the proliferative effect of miR-96 on breast cancer cells. Likewise, when MCF-7 cells were simultaneously transfected with miR-96 mimic and the PTPN9-overexpression plasmid, PTPN9 dramatically attenuated the promotive effect of miR-96 on cell migration in wound healing assay (Fig. 8E and F). In addition, co-transfection of miR-96 mimic and the PTPN9-overexpression plasmid markedly decreased the number of MCF-7 cells that passed through the transwell chamber (Fig. 8G and H), indicating that miR-96-resistant PTPN9 is sufficient to reverse the pro-invasion effect of miR-96 on breast cancer cells. Taken together, these results indicate that miR-96 may regulate the proliferation, migration and invasion of breast cancer cells through a PTPN9-dependent manner.

## Discussion

Over the past decade, many researchers have reported the dysregulation of miRNAs in the initial and developmental stages of human cancers. Given the abundant expression of miRNAs in cancers, the correction of cellular miRNA levels may emerge as a potential therapeutic strategy. Overexpressed miRNAs can be silenced using antagomirs, and miRNAs that are lost in cancers can be re-expressed using miRNA mimics. Indeed, some scientists have already established the potential usefulness of miRNAs as therapeutic molecules against cancers, including the inhibition of cancer cell proliferation by miR-26a in a mouse model of hepatocellular carcinoma and the prevention of metastasis formation by silencing of miR-10b^31^,^32^. In this study, we validated that miR-96 is upregulated in many breast cancer tissues and it could promote breast cancer cell proliferation, migration and invasion *in vitro* and tumor growth *in vivo*. Thus, it is quite possible that treatment with miR-96 antagomir may be a promising strategy for breast cancers showing upregulation of miR-96. In agreement with this hypothesis, miR-96 has also been shown to be upregulated in various human tumor types, including colorectal cancer, hepatocellular carcinoma and chronic myeloid leukemia cells^33^,^34^,^35^. It has also been reported that the suppression of miR-96 inhibited the proliferation and invasion of hepatocellular carcinoma cells^20^,^36^,^37^. Although many preclinical studies show the potential of miR-96 as a therapeutic target of human cancers, few clinical studies are currently underway. Greater research emphasis is needed to characterize the feasibility of targeting miR-96 in cancer therapy and develop simplified and cost-effective manipulation methods.

Protein tyrosine phosphatases (PTPs) are key regulators in various aspects of cellular functions, and dysregulation of PTPs is a major cause of human diseases, including cancers^38^. PTPN9 is a cytoplasmic phosphatase belonging to classic tyrosine-specific PTPs^30^,^38^,^39^. It has been reported that PTPN9 directs dephosphorylation of the ErbB family, including EGFR (epidermal growth factor receptor) and ErbB2, to inhibit receptor tyrosine kinase (RTK) activation^29^. Moreover, PTPN9 has also been shown to mediate dephosphorylation of STAT3 (signal transducer and activator of transcription 3)^30^. Because EGFR and ErbB2 play a very important role in the development of breast cancer, and constitutive STAT3 tyrosine or serine phosphorylation is frequently observed in breast carcinomas^40^,^41^, PTPN9 may contribute to tumor suppression by dephosphorylation of EGFR, ErbB2 and STAT3 and silencing of ErbB and STAT3 signaling pathways, thereby having potential therapeutic value for breast cancer. In this study, we showed that PTPN9 protein levels were significantly lower in breast cancer tissues and that PTPN9 had a negative effect on cell proliferation and invasion in breast cancer cells. Thus, PTPN9 may be a potential new target for breast cancer therapy. Technical limitations make it difficult to stably express PTPN9 *in vivo*. However, because miR-96 is an upstream regulator of PTPN9, it is possible to downregulate miR-96 for restoration of PTPN9 expression *in vivo*. Nevertheless, considerable further research must be performed to develop novel therapeutic strategies for breast carcinogenesis.

It is well known that a single miRNA can target multiple genes while multiple miRNAs can target a single gene. Thus, miR-96 may have multiple different mRNA targets other than PTPN9, and we could not rule out the possibility that additional targets are affected by miR-96 simultaneously. For example, miR-96 has been reported to directly repress the tumor suppressor genes *FOXO1* and *FOXO3a*^8^,^13^,^14^. Therefore, at this stage it is important to investigate how critical the newly identified pathway would be in the web of breast tumorigenesis. In this study, we found that miR-96 overexpression can promote the proliferation, migration and invasion of breast cancer cells and that PTPN9 reduction can mimic the miR-96-induced cellular phenotypes. More importantly, restoration of PTPN9 expression with a miR-96-resistant PTPN9 overexpression plasmid completely reversed miR-96-induced cellular phenotypes, suggesting that targeting PTPN9 is a major mechanism by which miR-96 exerts its oncogenic function. Therefore, modulation of PTPN9 by miR-96 may explain, at least in part, why the upregulation of miR-96 can promote cell proliferation and invasion and tumor growth in breast cancer.

Why miR-96 is upregulated during breast tumorigenesis is an interesting question deserving further discussion. STAT3 is a transcription factor that belongs to the STAT family. In response to cell stimuli, STAT3 is phosphorylated, forms homo- or heterodimer, and translocates to the cell nucleus where it acts as transcription activator^42^. STAT3 is constitutively activated or overexpressed in numerous cancer types, including more than 40% of breast cancers^43^,^44^,^45^. Interestingly, recent study has identified conserved STAT3 binding motifs in the promoter region of miR-183-96-182 cluster and showed that STAT3 directly binds to this site and increases miRNA cluster expression^46^. Thus, the observed upregulation of miR-96 in breast cancer may be due to activated STAT3. Because miR-96 negatively regulates PTPN9 expression, PTPN9 directly interacts with STAT3 and mediates its dephoshorylation^23^, while phosphorylated STAT3 promotes miR-96 expression^30^,^46^, miR-96, PTPN9 and STAT3 may form a double-negative feedback loop (equal to positive feedback) controlling cellular phenotypes in breast cancer. Because positive feedback is known to amplify a response into a self-sustained mode that is autonomous to the original stimuli, it is tempting to speculate that, once this feedback loop forms, PTPN9 expression will be maintained at a low level, and the miR-96-mediated cell function allows breast cancer cells to become more autonomous, e.g., to reproduce more rapidly and metastasize to new microenvironments.

In conclusion, this study demonstrates for the first time that miR-96 possesses oncogenic activity by negatively regulating PTPN9 expression in breast cancer. Further research on miR-96 and PTPN9 may reveal a new avenue for treatment of breast cancer.

## Additional Information

**How to cite this article**: Hong, Y. *et al*. miR-96 promotes cell proliferation, migration and invasion by targeting PTPN9 in breast cancer. *Sci. Rep*. **6**, 37421; doi: 10.1038/srep37421 (2016).

**Publisher's note**: Springer Nature remains neutral with regard to jurisdictional claims in published maps and institutional affiliations.

## Supplementary Material

Supplementary Information
