# Supplementary material for: miR-96 promotes cell proliferation, migration and invasion by targeting PTPN9 in breast cancer
Source: Sci Rep. 2016 Nov 18;6:37421. doi: 10.1038/srep37421 (PMC5114647; doi:10.1038/srep37421)
Supplement: Supplementary Information [file srep37421-s1.doc]

**Supplementary materials**

**miR-96 promotes cell proliferation, migration and invasion by targeting PTPN9 in breast cancer**

Yeting Hong, Hongwei Liang, Uzair-ur-rehman, Yanbo Wang, Weijie Zhang, Yong Zhou, Song’an Chen, Mengchao Yu, Sufang Cui, Minghui Liu, Nan Wang, Chao Ye, Chihao Zhao, Yanqing Liu, Qian Fan, Chen-Yu Zhang, Jianfeng Sang, Ke Zen, Xi Chen

**Supplementary Tables**

**Supplementary Table 1.** **Patients’ Characteristics**

| Case No. | Clinical History | Gender | Age (years) | TNM Stage |
| --- | --- | --- | --- | --- |
| BC #1 | IDC | Female | 56 | II |
| BC #2 | IDC | Female | 58 | II |
| BC #3 | IDC | Female | 60 | II-III |
| BC #4 | IDC | Female | 56 | III |
| BC #5 | IDC | Female | 58 | I-II |
| BC #6 | IDC | Female | 49 | II |
| BC #7 | IDC | Female | 50 | II-III |
| BC #8 | IDC | Female | 55 | II-III |
| BC #9 | IDC | Female | 48 | I-II |
| BC #10 | IDC | Female | 52 | II-III |

**Supplementary Table 2. The sequences of primers**

| PTPN9 (sense) | 5’-CCTGCCTTAGACTGGGACT-3’ |
| --- | --- |
| PTPN9 (antisense) | 5’-TTCGCTTTGTTAGCTTCACT-3’ |
| Cyclin D1 (sense) | 5’-AGCTGTGCATCTACACCGAC-3’ |
| Cyclin D1 (antisense) | 5’-TGTGAGGCGGTAGTAGGACA-3’ |
| CDK6 (sense) | 5’-GGATAAAGTTCCAGAGCCTGGAG-3’ |
| CDK6 (antisense) | 5’-GCGATGCACTACTCGGTGTGAA-3’ |
| CDK4 (sense) | 5’-TGGTGTCGGTGCCTATGGGA-3’ |
| CDK4 (antisense) | 5’-ACGGGTGTAAGTGCCATCTG-3’ |
| p21 (sense) | 5’-GCGGAACAAGGAGTCAGA-3’ |
| p21 (antisense) | 5’-GGAGAAACGGGAACCAG-3’ |
| GAPDH (sense) | 5’-CGAGCCACATCGCTCAGACA-3’ |
| GAPDH (antisense) | 5’-GTGGTGAAGACGCCAGTGGA-3’ |

**Supplementary Figures**

**
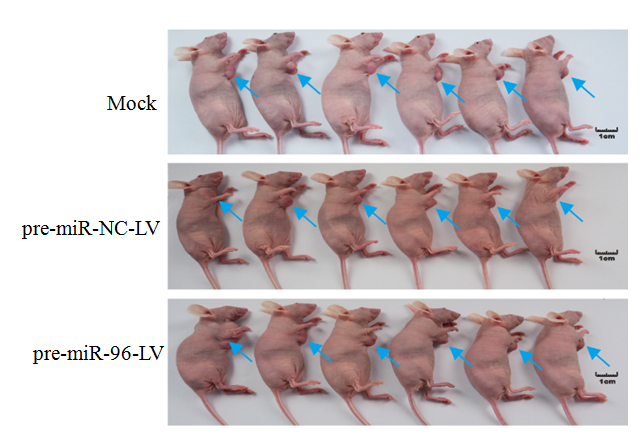
**

**Supplementary Figure 1. Images of the nude mice bearing xenograft tumors.** MCF-7 cells were infected with a control lentivirus (pre-miR-NC-LV) or a lentivirus to overexpress miR-96 (pre-miR-96-LV) and then implanted subcutaneously into 4-week-old nude mice. Tumor growth was evaluated at day 21 after cell implantation. Mice implanted with wide-type MCF-7 cells (Mock) serve as the negative control.

**
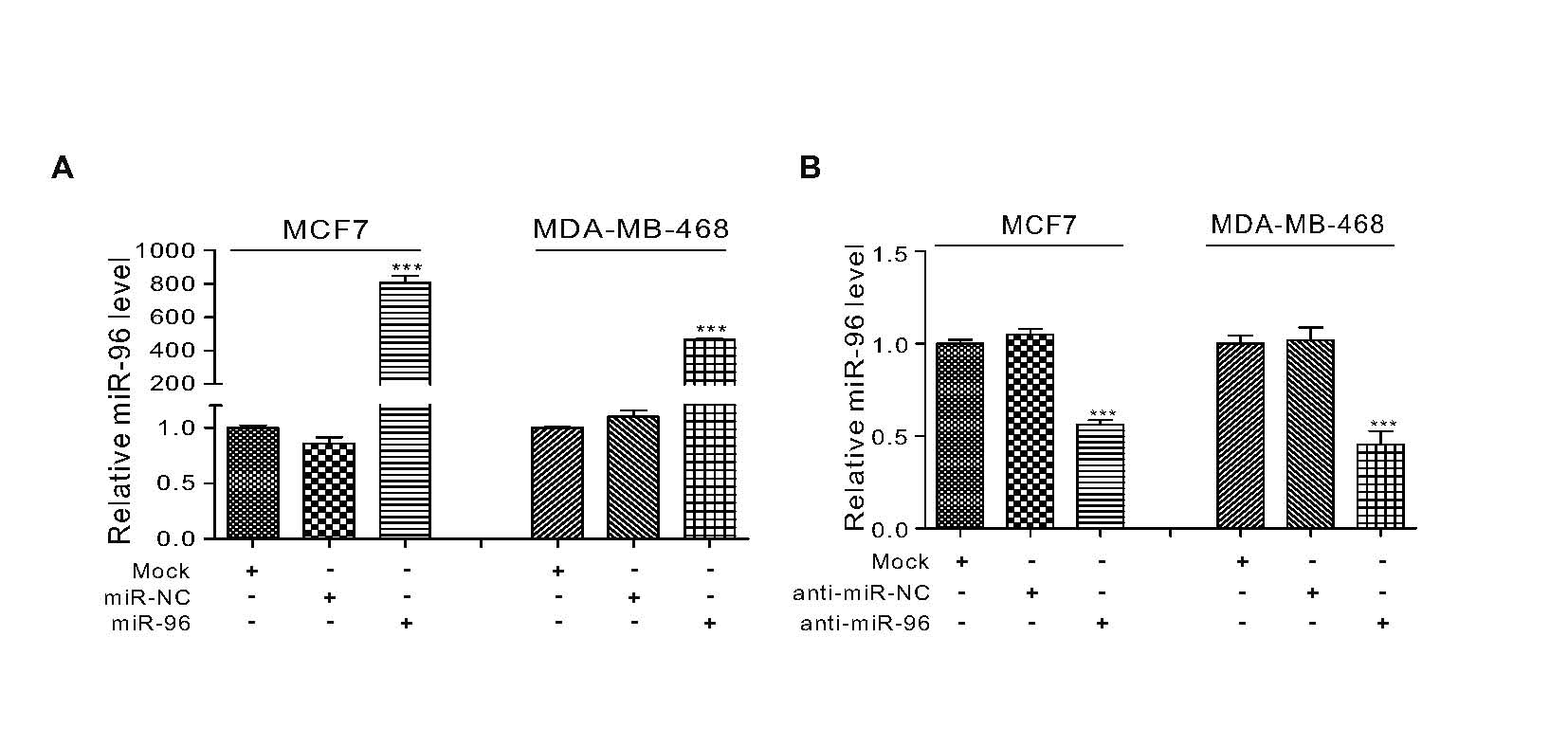
**

**Supplementary Figure 2. Evaluation of the overexpression and knockdown efficiency of miR-96. (A)** Quantitative RT-PCR analysis of miR-96 levels in MCF-7 and MDA-MB-468 cells after overexpression of miR-96. **(B)** Quantitative RT-PCR analysis of miR-96 levels in MCF-7 and MDA-MB-468 cells after knockdown of miR-96. ***P<0.001.

**
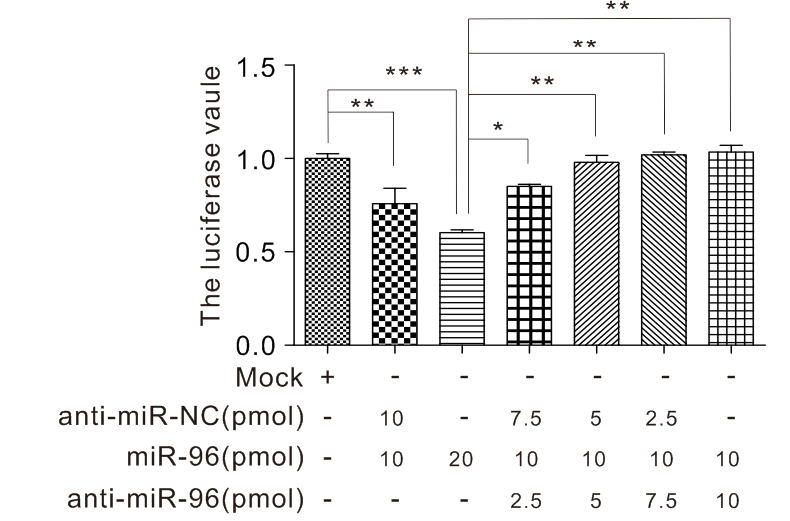
**

**Supplementary Figure 3. The effect of anti-miR-96 on miR-96-induced reduction of luciferase reporter activity.** Firefly luciferase reporters containing miR-96 binding sites in the PTPN9 3’-UTR were co-transfected into 293T cells with different doses of miR-96, anti-miR-NC or anti-miR-96. The cells transfected only with luciferase reporters (Mock) serve as the negative control. Twenty-four hours after transfection, luciferase assays were performed. Firefly luciferase values were normalized to β-galactosidase activity and the results were calculated as the ratio of firefly luciferase activity in the transfected cells normalized to the mock cells. *P < 0.05; **P < 0.01; ***P<0.001.


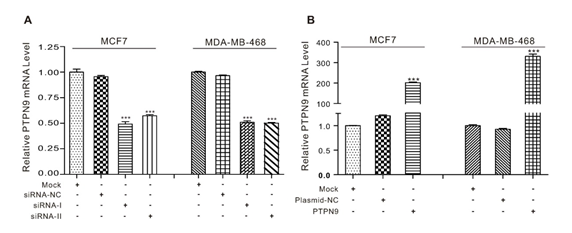


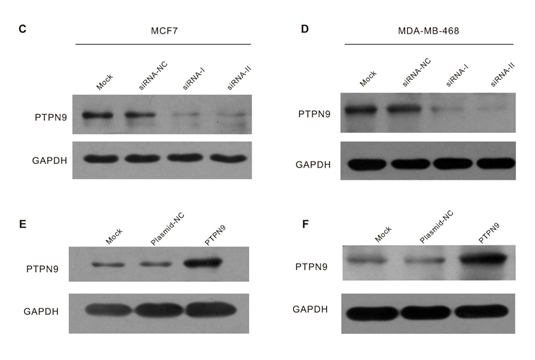


**Supplementary Figure 4. Evaluation of the overexpression and knockdown efficiency of PTPN9. (A and B)** Quantitative RT-PCR analysis of PTPN9 mRNA levels in MCF7 and MDA-MB-468 cells transfected with equal doses of siRNA-NC or PTPN9 siRNA, or with equal doses of plasmid-NC or PTPN9 overexpression plasmid. **(C and D)** Western blotting analysis of PTPN9 protein levels in MCF7 and MDA-MB-468 cells treated with transfected with equal doses of siRNA-NC or PTPN9 siRNA. **(E and F)** Western blotting analysis of PTPN9 protein levels in MCF7 and MDA-MB-468 cells transfected with equal doses of plasmid-NC or PTPN9 overexpression plasmid.
